# Chemical Profiling and Quality Assessment of Food Products Employing Magnetic Resonance Technologies

**DOI:** 10.3390/foods14142417

**Published:** 2025-07-09

**Authors:** Chandra Prakash, Rohit Mahar

**Affiliations:** Department of Chemistry, Hemvati Nandan Bahuguna Garhwal University (A Central University), Srinagar Garhwal 246174, Uttarakhand, India

**Keywords:** magnetic resonance, metabolomics, adulteration, foodstuffs, magic-angle spinning

## Abstract

Nuclear Magnetic Resonance (NMR) and Magnetic Resonance Imaging (MRI) are powerful techniques that have been employed to analyze foodstuffs comprehensively. These techniques offer in-depth information about the chemical composition, structure, and spatial distribution of components in a variety of food products. Quantitative NMR is widely applied for precise quantification of metabolites, authentication of food products, and monitoring of food quality. Low-field ^1^H-NMR relaxometry is an important technique for investigating the most abundant components of intact foodstuffs based on relaxation times and amplitude of the NMR signals. In particular, information on water compartments, diffusion, and movement can be obtained by detecting proton signals because of H_2_O in foodstuffs. Saffron adulterations with calendula, safflower, turmeric, sandalwood, and tartrazine have been analyzed using benchtop NMR, an alternative to the high-field NMR approach. The fraudulent addition of *Robusta* to *Arabica* coffee was investigated by ^1^H-NMR Spectroscopy and the marker of *Robusta* coffee can be detected in the ^1^H-NMR spectrum. MRI images can be a reliable tool for appreciating morphological differences in vegetables and fruits. In kiwifruit, the effects of water loss and the states of water were investigated using MRI. It provides informative images regarding the spin density distribution of water molecules and the relationship between water and cellular tissues. ^1^H-NMR spectra of aqueous extract of kiwifruits affected by elephantiasis show a higher number of small oligosaccharides than healthy fruits do. One of the frauds that has been detected in the olive oil sector reflects the addition of hazelnut oils to olive oils. However, using the NMR methodology, it is possible to distinguish the two types of oils, since, in hazelnut oils, linolenic fatty chains and squalene are absent, which is also indicated by the ^1^H-NMR spectrum. NMR has been applied to detect milk adulterations, such as bovine milk being spiked with known levels of whey, urea, synthetic urine, and synthetic milk. In particular, T2 relaxation time has been found to be significantly affected by adulteration as it increases with adulterant percentage. The ^1^H spectrum of honey samples from two botanical species shows the presence of signals due to the specific markers of two botanical species. NMR generates large datasets due to the complexity of food matrices and, to deal with this, chemometrics (multivariate analysis) can be applied to monitor the changes in the constituents of foodstuffs, assess the self-life, and determine the effects of storage conditions. Multivariate analysis could help in managing and interpreting complex NMR data by reducing dimensionality and identifying patterns. NMR spectroscopy followed by multivariate analysis can be channelized for evaluating the nutritional profile of food products by quantifying vitamins, sugars, fatty acids, amino acids, and other nutrients. In this review, we summarize the importance of NMR spectroscopy in chemical profiling and quality assessment of food products employing magnetic resonance technologies and multivariate statistical analysis.

## 1. Introduction

Nuclear Magnetic Resonance (NMR) Spectroscopy is a powerful and versatile analytical tool for determining the structure of complex compounds [1,2]. NMR utilizes the magnetic behavior of certain atomic nuclei (i.e., ^1^H, ^2^H, ^13^C, ^31^P, ^19^F, ^15^N), which possess nuclear spin and behave like tiny magnets. When these nuclei are placed in a strong external magnetic field, they align with or against the magnetic field, creating discrete spin states that have different energy levels. The nuclei precess around the magnetic field at a characteristic precessional frequency (also known as Larmor frequency), which is unique for each of the nuclei and the strength of the applied magnetic field [3]. The application of radiofrequency (RF) creates a transition from a lower to a higher energy, while matching the energy difference between nuclear spin states. During the relaxation process, nuclei come back to the lower energy state and emit RF signals in the form of Free Induction Decay (FID), which is Fourier transformed into a frequency domain signal to gain information about the molecular environments. The electronic environment influences the magnetic field of nuclei through nuclear shielding and deshielding, causing slight variations in resonance frequencies called chemical shifts. These characteristic shifts have helped the structural elucidation of molecules in pure form and even in complex mixtures [4]. NMR instrumentation includes a strong superconducting magnet (usually for high-field NMR) that generates the homogeneous magnetic field necessary for generating nuclear spin states [5] and a radiofrequency system (RF coils or probes) that transmits RF pulses to excite the nuclei and receives the tiny emitted signals [6]. Additionally, the instrumentation includes detectors and receivers that capture the NMR signals, and a software system for data acquisition, overall system control, and spectral analysis [7].

NMR has not only been used to elucidate the structure of compounds in pure form but also has the potential to reveal the composition of mixtures as well as their native states [8,9,10]. High-resolution NMR provides insight into the chemical structure of isolated purified compounds or compounds in complex mixtures [11]. NMR is a non-destructive technique that provides key information about the chemical makeup, structure, and arrangement of components in food items. ^1^H NMR spectroscopy has been used for identifying adulteration of olive oil with low-quality hazelnut oils. NMR spectra revealed that, in hazelnut oils, linolenic acid and squalene are almost absent [12]. Some of the other studies include the following: ^1^H-NMR analysis of extracts of kiwifruits affected by disease, i.e., elephantiasis, revealed a higher number of small oligosaccharides than in healthy fruits [13]. A proton time domain NMR experiment has been employed to detect adulteration in milk, such as bovine milk being mixed with whey, urea, hydrogen peroxide, synthetic urine, and milk. The increment in spin–spin (T2) relaxation time was observed in adulterated milk with respect to pure milk [14]. Another method, namely cross-polarization magic-angle spinning (CP-MAS), has evolved for the analysis of solid samples. It combines fast rotation of the sample at a magic angle, decoupling of protons, and cross-polarization (transferring proton magnetization to a heteronuclear (^13^C)) to enhance its sensitivity [15]. CP-MAS with ^13^C has been employed for the analysis of insoluble food samples such as fibers and proteins. Additionally, the solid-state NMR technique was also employed along with other NMR active nuclei such as ^31^P and ^2^H for investigating the interactions between lipids and bioactive compounds present in foodstuffs [16]. Low-field (LF) NMR spectroscopy is available in benchtop-type NMR spectrometers operating at low magnetic fields of 1–2 Tesla (40–80 MHz), and using a permanent compact magnet instead of superconducting magnets like in high-field NMR [17]. The data acquisition and processing are similar to high-field NMR, but the resolution and sensitivity are quite low in the case of LF-NMR. Low-field ^1^H-NMR relaxometry is an important technique for analyzing the essential components of whole food items using relaxation parameters, self-diffusion of water, and strength of NMR signals [18]. Quantitative Nuclear Magnetic Resonance (qNMR) provides insight into food analysis and has been employed for precise quantification of key metabolites and verification of food authenticity [13]. Benchtop NMR has provided information about saffron adulteration by analyzing substances including safflower, turmeric, cayenne pepper, and tartrazine. This could be an alternative approach to the other NMR method [19]. The fraudulent addition of Robusta to Arabica coffee has been detected by the ^1^H-NMR spectrum of the extract of coffee powder, demonstrating its application in measuring food adulteration [20].

The basic physics and principles behind MRI technology are similar to the NMR spectrometer, but the sampling of MRI data and reconstruction of the two-dimensional and three-dimensional images of objects are the main features of MRI instrumentation. The signal of each voxel (volume element) depends on the physical properties, i.e., proton spin density, relaxation times, diffusion, and local differences in magnetic susceptibility [21,22].

High-resolution MRI (also known as microscopic MRI (µMRI)) can produce images with a high resolution in micron pixel size [23,24,25,26], opening a new avenue to explore MRI protocols in the agriculture sector for analyzing roots, plant parts, fruits, and vegetables [27,28]. This high-resolution MRI has been used efficiently to investigate the morphological differences in kiwifruit. The T2-weighted images provide information about water loss, which induces cellular interactions, causing morphological changes and softening [29]. The high-resolution magic-angle spinning (HRMAS) NMR technique has been developed for analyzing components in matrices and intact semi-solid samples [30], producing high-resolution NMR spectra [31]. The advancement of HRMAS and high-resolution microscopic MRI could circumvent the pretreatment of the sample, and the standard protocols can be developed for rapid measurements. These MR-based techniques have the potential to be effortlessly transitioned into quality control applications in the food industry. The HRMAS NMR technique is a very effective and recent advancement in the field of NMR for analyzing semi-solid and gel-like samples [32]. The HRMAS NMR technique utilizes the concept of the “magic angle”, in which the orientation of the sample containing the NMR probehead is at a specific angle (54.74°) relative to the external magnetic field [33]. This technique provides better resolution for semi-solid samples via rotation of samples at a magic angle to minimize orientation-dependent (or dipole–dipole) interactions. Further, this technology provides partial averaging of the anisotropic interactions, minimizing their influences on line broadening, resulting in higher resolution (i.e., narrower lineshape) in the observed NMR spectra [34]. HRMAS techniques minimize anisotropic processes through rapid spinning of samples at a magic angle, enhancing spectral quality. This method can simultaneously analyze polar and non-polar components without requiring sample extraction [35].

NMR spectroscopy, along with multivariate statistical analysis, can be employed for monitoring changes in food constituents and assessing self-life and the impact of weather and storage conditions over a period of time. NMR can be an invaluable technique for the quantification of vitamins, fatty acids, and amino acids, providing a comprehensive analysis of the nutritional profiles of foodstuffs [13]. Multivariate statistical analysis is an indispensable technique for analyzing multiple variables and a large number of observations at the same time [36]. There are several ways of performing multivariate analysis. Principal Component Analysis (PCA) is an unsupervised technique that reduces the dimensions of complex datasets by converting correlated variables into principal components [37]. The PLS-DA technique is a supervised technique that is particularly useful for multicollinear datasets, where the predictor variables are strongly correlated with each other [38]. PLS-DA is commonly employed to build predictive models for quantitative responses and discriminate between different classes or groups within the datasets. The method works by finding the linear combinations (or latent variables) of the original predictor variables, which helps explain the variance in the response variable, ultimately maximizing the separation between the classes [39]. Orthogonal Projections to Latent Structures Discriminant Analysis (OPLS-DA) is another powerful supervised multivariate statistical analysis technique that aims to separate the variation in the data into the response variable (also known as class membership) and its orthogonal part [40]. OPLS-DA has been widely used for sample classification and predictive modelling [41,42]. Unsupervised PCA maximizes the variance in the data without taking the class information, but OPLS-DA explicitly models and demonstrates the differences between the classes.

In this review, we have summarized the various advancements in Magnetic Resonance technologies and chemometrics and their applications in chemical profiling and quality assessment of food products.

## 2. Extraction Methods

**Figure 1:**
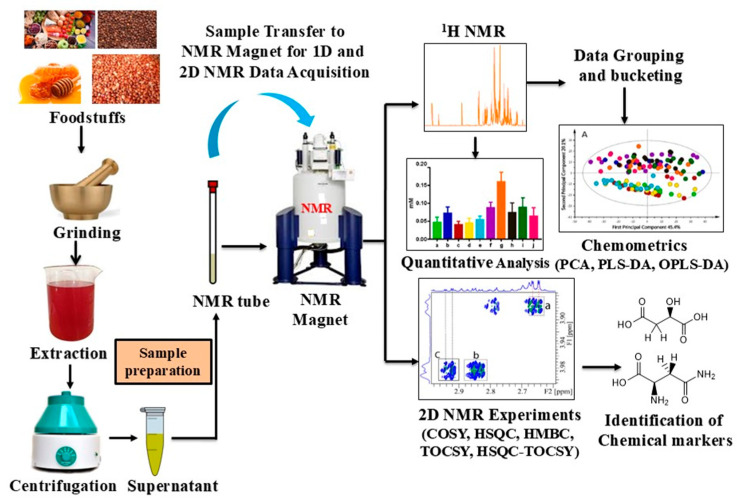
Procedure of the chemical profiling of foodstuffs using solution-state NMR spectroscopy and multivariate statistical analysis. Chemical profiling of foodstuffs using solution-state NMR involves griding (powdering) and solvent extraction of metabolites with variable polarity of solvents and analyzing them after adding deuterated solvents via high-resolution ^1^H-NMR probe. Processed spectra are binned, normalized, and statistically analyzed using PCA or PLS-DA to detect patterns and classify samples. Key metabolites are identified as chemical markers through 2D-NMR experiments along with multivariate models.

Solution-state NMR is the most common method of obtaining high-throughput NMR data for multivariate statistical analysis, and it is a widely available, affordable, and popular technique for NMR data acquisition. Lee et al. used dried and ground red peppers to enhance the extraction efficiency of the metabolites. Briefly, the sample (500 mg) was sonicated in 1200 μL of deuterated methanol and 400 μL of deuterated water for 30 min. Then the mixture was centrifuged for a few minutes at 13,500 rpm, and the supernatant was filtered and transferred to a 5 mm NMR tube for NMR analysis [43]. The rhizomes of *Curcuma longa* were collected, cleaned, chopped into pieces, and air-dried. The rhizomes were ground into powder, which was used for extraction. The *C. longa* samples were adulterated by adding *Curcuma manga* in various proportions. Curcumin standard solution (1 mg/mL) was produced using methanol. For sample preparation, 50 mg of *C. longa* powder was weighed, and 1.5 mL of methanol was added to it and vortexed for 5 min, followed by centrifugation for 5 min at 4000 rpm. The air-dried coriander seeds (1 g) were ground into powder, and metabolite extraction was performed using 10 mL of acetonitrile and water (1:1), vortexed, and incubated in an ice-water bath. Cell disruption was performed using ultrasonication (20 Hz, 20 s) for 15 min, followed by centrifugation at 16,000 rpm for 15 min at 4 °C. This procedure was repeated three times, and the supernatants were lyophilized to obtain a final dried powder of 30 mg [44]. Oils can be extracted using the milling method to minimize thermal degradation. Twenty-gram quantities of the sample were frozen in liquid nitrogen, ground into a paste, and stored overnight at 4 °C. Four milliliters of water were added to the paste, followed by centrifugation. The oil was collected from the upper layer and stored in amber vials until further analysis [45]. The above-mentioned extraction methods can be generalized for sample processing for solution-state NMR analysis of various foodstuffs. The procedure of solution-state NMR for quality assessment of foodstuffs is shown in Figure 1.

**Figure 2:**
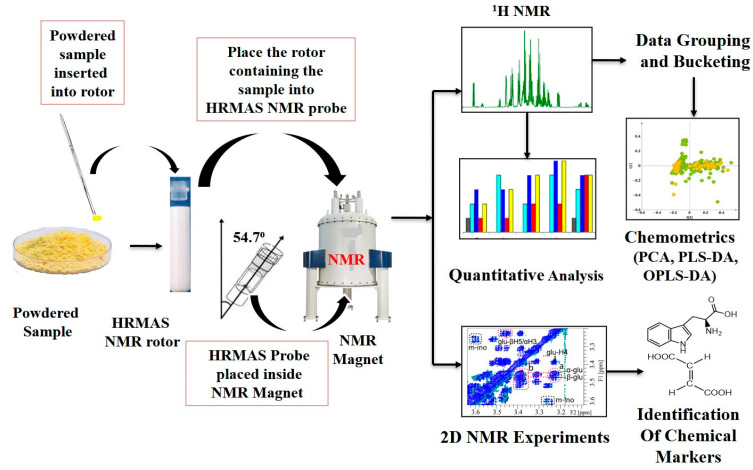
Procedure of the chemical profiling of foodstuffs using HRMAS NMR spectroscopy and multivariate statistical analysis. Chemical profiling of foodstuffs using HRMAS NMR involves analyzing intact or semi-solid food samples without extensive extraction. Samples are spun at the magic angle to obtain high-resolution spectra of complex matrices. Spectral data are processed, normalized, and subjected to multivariate analysis like PCA or PLS-DA for classification. Additionally, chemical markers can be identified through 2D-NMR experiments along with multivariate models.

HRMAS NMR and MRI experiments generally need very minimal sample preparation, as the sample can often be used in its original state. In the MRI technique, the originality of the foodstuffs remains the same, as no sample preparation is needed. This is very helpful as it allows recording of internal images of the food samples and can help in assessing the food quality and distribution of the metabolites (markers). In the HRMAS NMR technique, solid and semi-solid samples can be placed in the rotor (sample holder), and a small amount of deuterated solvent added to lock the magnetic field, which compensates for magnetic field drift during the data acquisition period. This technique primarily records the NMR spectra of samples (solid or semi-solid) with high resolution due to sample rotation at magic angle (54.74°), diminishing anisotropic or dipole–dipole interactions and providing significantly narrow lines (signals) (Figure 2) [46]. This facilitates the qualitative, quantitative, and multivariate analysis effectively. On the other hand, solution-state NMR requires significant treatment of the samples, starting from the extraction of components present in the foodstuffs to minimize the complexity (matrix effect) and facilitate identification and quantification of a large number of components (metabolites). Deuterated solvent is also added to the sample to lock the magnetic field.

## 3. Sample Preparation for NMR/MRS and MRI Analysis

Different types of food samples require different NMR sample preparation methods. For juice samples, 5 mL of each sample needs to be freeze-dried. Each freeze-dried sample (~200 mg) is shaken with 1.0 mL of deuterium oxide (D_2_O) and centrifuged at 12,000 rpm for 10 min. The supernatant is transferred to a 5 mm NMR tube, and sodium salt of trimethylsilylpropionic *2*, *2*, *3*, *3-d*_4_ sulfonic acid (TSP) is added as an internal reference (Vogels et al., 1996). Milk or milk-derived products can be dissolved in deuterated methanol containing 0.5% tetramethyl silane (TMS) and mixed well using a vortex mixer, followed by sonication, and equilibrated at room temperature for at least 12 h. After that, the mixture is centrifuged to remove insoluble components, and the supernatant is transferred into an NMR tube for analysis [47]. Diverse wine samples are prepared for NMR analysis by adding 100 μL of phosphate buffer (1 M KH_2_PO_4_ with 0.1% TSP, D_2_O, and NaN_3_) to 900 μL of wine. The pH should be adjusted to around 3.10 using bis(trifluoromethyl) phosphoric acid. From this solution, 600 μL is transferred into an NMR tube [48].

For HRMAS analysis, 25 mg of sweet pepper or other samples were placed in a 4 mm HRMAS rotor with a 50 μL spherical insert, and subsequently, 25 μL of phosphate buffer (0.01 M, 0.5% TSP, pH 7.2, D_2_O) was added to the sample [49]. The general procedure of HRMAS NMR for various samples in their intact state is depicted in Figure 2. Adulterated sesame oil samples were placed in a 50 mL screw-capped vial, vortexed at room temperature, and used for relaxation measurement with NMR [50]. In another study, pure or blended camellia oil (200 μL) was dissolved in deuterated chloroform (800 μL of CDCl_3_ containing 0.3% *v*/*v* TMS) for NMR sample preparation. Subsequently, 600 μL of this solution was transferred to an NMR tube for further analysis [51].

## 4. Data Acquisition in NMR and MRI

NMR and MRI data acquisition requires optimization of key parameters, which are sample-dependent, as the salt and water concentrations vary from sample to sample. Careful selection of experiments is necessary, such as presaturation (nullifying) of solvent signals for enhanced metabolites and lipids analysis, sufficient relaxation of the magnetization, improved signal-to-noise ratio (SNR), and resolution to achieve maximum information. The number of scans (NS) is an important parameter for detection and quantitative measurements of analytes present in the sample. The noise in the detection circuits is the primary source of noise in NMR, and the SNR scales as the square root of the NS is added [52]. The acquisition time is the time that is set for the signal detection time. As the Free Induction Decay (FID) is a decaying signal, acquisition time should be sufficiently longer (typically it is 1–5 s in ^1^H-NMR experiments) to obtain the maximum information [53]. The additional relaxation delay also needs to be optimized for sufficient relaxation of the net magnetization through T1 and T2 relaxation processes [54]. The total time taken, i.e., repetition time between the acquisition of the first scan and start of acquisition of the second scan, should be sufficiently long to recover the maximum signal, thereby enhancing the amplitude of the NMR signal [55]. Changing these parameters will change the quality of the NMR spectra, requiring careful optimization and a literature survey before NMR analysis of any foodstuffs.

Similarly, in MRI experiments, the molecular motion governs the T1 and T2 relaxation times in food samples, as the complexity of the tissue organization leads to the different compartments of the water molecules [56]. The basic pulse sequences, such as spin echo (single and multi-slice), gradient echo, saturation, and inversion experiments, are the key experiments for food analysis [57]. Slice orientation, slice thickness, field of view, and encoding steps contribute to the SNR and resolution of the images [58]. To demonstrate the set of parameters required for NMR and MRI experiments, the relevant information is summarized from some of the studies.

Proton NMR spectra of polar peanut extract were acquired on a Bruker 400 MHz spectrometer employing the noesygppr1d pulse sequence for water suppression. The spectra were recorded at 300 K with 128 scans, 65,536 complex data points, and a spectral width of 8417.5 Hz. Additionally, ^1^H NMR spectra of the extract were obtained for marker identification on a 600 MHz spectrometer using the zgesgp pulse sequence. The relaxation delay (D1) was set to 60 s for quantitative analyses [59]. In one of the studies, a pulsed field gradient diffusion NMR experiment was performed for the assessment of extra-virgin olive oil using a Bruker 300 MHz spectrometer equipped with an inverse probe capable of providing a Z gradient strength of 350 G/cm. To prevent probe heating, the probe was continuously cooled with water to protect the sample from heating. Diffusion coefficients were calculated from the monoexponential decay of the signal with gradient amplitude. A standard calibration sample (D_2_O, D = 1.872 × 10^9^ m^2^/s) was used before each set of experiments to ensure consistency across the samples [60]. One-dimensional and two-dimensional NMR data of authentic and adulterated honey samples were recorded using a Bruker 400 spectrometer. The presaturation technique with acquisition parameters of data points = 16 K; delay time = 10 s; acquisition time = 1.71 s; number of scans, 8; and spectral width of 2003.205 Hz. For the heteronuclear multiple bond correlation (HMBC), the NMR spectrum was obtained by ^1^H-^13^C correlations via heteronuclear quantum coherence. This method is used to enhance the detection of long-range couplings ^1^H-^13^C. [61]. The contaminated milk sample was studied using ^1^H TD-NMR experiments in an NMR spectrometer equipped with a permanent magnet of 0.23 T. Transverse relaxation time (T2) was calculated using a (Carr–Pursell–Meiboon–Gill (CPMG)) pulse sequence. The key parameters were kept at a 90° pulse width of 16 μs, echoes of 400 μs, and a 15 s recycle delay [14]. In another work, ^1^H NMR spectra were recorded at 600.38 MHz on a Bruker 600 spectrometer using a cryoprobe and Z gradient. The NMR raw data were processed in MestReNova. Chemical shifts were calibrated using TMS as an internal reference at 0.00 ppm. The spectra were converted into buckets over the spectral width from 10 to 0.5 ppm, excluding the residual solvent signal in the MestReNova. The constant bucketing width of 0.01 ppm is commonly used in NMR metabolomics, while the milk sample used a width of 0.26 ppm, since it coincided with the larger peaks of fatty acyl chains [51]. The Free Induction Decays (FIDs) of peanuts were Fourier transformed (FT) with a 0.3 Hz line broadening factor. The spectra generated were calibrated to the TMSP signal, and automatic phase and baseline correction were applied. The signal-to-noise ratio was determined using the implemented *sino* command in TopSpin [59].

The ^1^H-NMR spectra of cinnamon spice were Fourier transformed to ESP files using ACD/NMR software (https://www.acdlabs.com/products/, accessed on 30 June 2025) (Toronto, Canada). The NMR spectra were referenced to internal standard hexamethyl disiloxane (HMDS) at 0.062 ppm and 1.96 ppm for ^1^H and ^13^C NMR, respectively. Spectral intensities were reduced to integrated regions (buckets) of equal width (0.04 ppm) within the region of δ 11.4 to 0.4 ppm. The residual water and methanol regions were removed before the multivariate analysis [62].

Low-field NMR was used to measure transverse relaxation times with a working frequency of 11 MHz and a 15 mm-diameter probe. The T2 relaxation time was measured using the CPMG pulse sequence with the following pulse parameters: center frequency = 200 kHz, recycle time = 3500 ms, and echo count = 4500. The CPMG data were processed using an exponential function to obtain discrete T2, and a linear regression analysis was conducted using Origin software (https://www.originlab.com/, accessed on 30 June 2025) [50]. ^1^H-NMR data of edible oils were obtained using a 600 MHz Bruker NMR spectrometer. The pulse sequence, zg30, includes a relaxation delay time of 1 s, an acquisition time of 4.95 s, and a spectral width of 6613.8 Hz. The FIDs were multiplied by a function of 0.5 Hz line width for increasing SNR on the MestReNova software (https://mestrelab.com/, accessed on 30 June 2025). The phase was adjusted, and the baseline was corrected using a multipoint base correction function [63].

**Figure 3:**
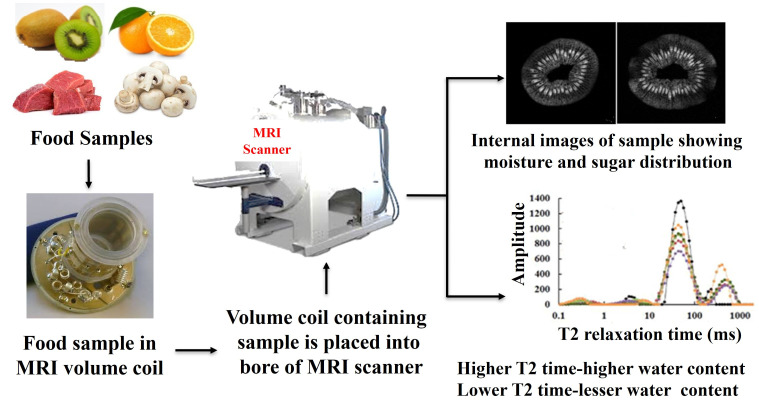
Procedure of MRI-based investigation of the internal quality, structure, and composition of food samples. The MRI technique non-invasively examines internal structure (tissue organization), moisture distribution, and composition of food samples. It provides high-resolution images to illustrate the quality, texture, and uniformity over the entire region without damaging the product.

Imaging technologies can be considered the best technique to visualize the internal tissue organization and distribution of the metabolites in the food samples. The choice of magnet, the probehead (or MRI resonator) for various NMR active nuclei with different inner diameters, and imaging protocols are the essential components required for MRI data acquisition (Figure 3). The food samples can be cut into small pieces and fit into the small sample tubes for variable-size resonators. The axial, sagittal, and coronal images can be taken utilizing various MRI protocols. These images help in understanding the inner distribution of the components present in food samples.

Typically, the water signal is monitored and used for image reconstruction using Gradient-echo (GE) and multi-slice multi-echo (MSME) experiments. The images generated using MRI provide information about the interaction of macromolecules with water, generating multicomponent water molecules inside the food samples. T2-weighted (relaxation time-based) MRI images provide information about the amount and binding of water molecules inside the samples [64]. Additionally, diffusion-weighted MRI experiments can be used to measure diffusion coefficients in food samples, and this can delineate fiber orientation and molecular distribution in the foodstuffs [65,66]. Low-field NMR and MRI experiments can demonstrate the effect of fatty acid distribution and surface morphology of food samples containing oils. For example, Yang et al. utilized a potato strip and wrapped it with tape to prevent contamination of the probe. Coronal slice was selected as the imaging orientation, and the test resonance frequency was set at 23.31 MHz, and recorded NMR and MRI data [67].

## 5. Statistical Analysis of NMR Data

Statistical analysis of ^1^H NMR data of red pepper powders was conducted using SPSS Statistics software (https://www.ibm.com/products/spss, accessed on 30 June 2025). The significant differences in metabolites between various types of pepper were determined using analysis of variance (ANOVA). For the signals with significant F-values, which indicate the large variations between group, means were calculated to quantify the variation within the group. Duncan’s multiple-range test (*p* < 0.05) was performed to confirm the difference between the means [43]. The data were obtained in triplicate and presented as mean ± standard deviation (SD). Unsupervised Principal Component Analysis (PCA) and K-means cluster distribution tests have been applied to analyze the dataset of black pepper (*Piper nigrum*) using SPSS software [68]. The ^1^H NMR complex datasets of *C. manga* and *C. longa* have been subjected to multivariate analysis using Minitab (https://www.minitab.com/en-us/products/minitab/, accessed on 30 June 2025) and SIMCA software (https://www.sartorius.com/en/products/process-analytical-technology/data-analytics-software/mvda-software/simca, accessed on 30 June 2025). These species displayed variations in the chemical shift due to various metabolites. PCA, a form of multivariate analysis, was applied to differentiate between pure and adulterated powder of *C. longa* with *C. manga*. OPLS-DA is a supervised method that is quite useful for pattern recognition tools compared to PCA. OPLS-DA displayed a clear separation of *C. longa* and adulterated *C. longa* and demonstrated a good fit (R^2^ = 0.91) and good predictivity (Q^2^ = 0.71).

Binned ^1^H NMR data were subjected to multivariate statistical analysis to discriminate red grapes from white wines. The investigation of three grape varieties of red and white wines was performed and discrimination was achieved successfully using PCA. Internal leave-one-out cross-validation (LOOCV) was utilized to validate the PCA model. Red wine and white wine varieties provided LOOCV correct average classification rates of 82% and 94%, respectively [69]. NMR fingerprints of different plant species, such as *Cinnamomum verum* and *Cinnamomum cassia* species, can be observed by PCA. The PCA showed the distinctions between the two species (*C. verum* and *C. cassia*). The loading plot of metabolites presented the most significant components and the signals related to eugenol, cinnamaldehyde, and fatty acids, which are necessary for sample segregation. The OPLS-DA model identified the metabolic patterns of each species. The OPLS-DA score plot explained 77% of the total variance with a predictive accuracy of (R^2^ = 0.77, Q^2^ = 0.65). The loading plot illustrates that eugenol, distinguished in *C. verum* by OCH_3_ signals, signifies its superior quality as a spice, whereas, *C. cassia* is enriched with fatty acids, as indicated by signals shown by NMR and loading plot [62].

In another study, ^1^H TD-NMR in conjunction with chemometrics was employed to detect adulteration in milk. PCA was performed on the normalized TD-NMR relaxation decays. The samples that were adulterated with H_2_O_2_ were identified because of nonlinear T2 variation. PC1 demonstrates 73.2% of the variation, and control milk samples were grouped at one end, whereas adulterated samples were grouped at the other end of PC1 [14]. The quality parameters of sorghum juice were predicted using a multivariate regression model, which uses ^1^H TD-NMR, and indicated that the T2 is responsible for differences in data in the PCA model [70]. The regression models developed using full TD-NMR relaxation show better predictability than the univariate model derived from T2 values. These models have been used to quantify the adulteration of milk [71].

**Analysis of Variance (ANOVA):** ANOVA is a statistical test that uses analytical data such as NMR-derived concentration and peak (signal) intensities to compare the means of two or more groups to determine the significant differences between them for variables under study. ANOVA works by analyzing the variance within each group as well as between the groups [72].

**Multivariate Statistical Analyses:** Multivariate analyses such as PCA, PLS-DA, and OPLS-DA are frequently employed to deduce meaningful characteristic patterns from complex datasets obtained from NMR, LC-MS, GC-MS, and other analytical platforms. Each of the multivariate analysis methods is briefly described below:

**Principal Component Analysis (PCA):** PCA is an unsupervised method that effectively classifies different samples under study using the metabolites, with different components (PC1, PC2, and so on). Mostly, the first component explains the maximum variance and separation patterns. PCA clusters the sample type with robustness [73]. PCA strategically transforms correlated variables into linearly uncorrelated variables (known as principal components (PCs)) through orthogonal transformations. PCA compresses raw data into PCs to deduce the characteristics of the original dataset. PC1 embodies the most salient feature, with PC2 capturing the next most significant feature, and so on, in a multidimensional data matrix.

**Partial Least Squares Discriminant Analysis (PLS-DA):** PLS-DA is a supervised dimensionality reduction method, which is prevalent in chemometrics and highly recommended for omics data analysis. PLS-DA performs dimensionality reduction with the consideration of group information. PLS-DA facilitates dimensionality reduction, feature selection, and sample classification. Cross-validation is a model validation procedure that provides critical insights into the performance and robustness of the PLS-DA model. Cross-validation of the model is demonstrated by quality parameters R^2^ and Q^2^. R^2^ measures the goodness of fit, and a higher the value of R^2^ indicates a more perfect description of the data. Q^2^ measures the predictive ability, and a higher value represents perfect predictability. A large discrepancy between R^2^ and Q^2^ values suggests an overfitting of the model for given datasets through the use of many components [74].

**Orthogonal Partial Least Squares Discriminant Analysis (OPLS-DA):** It integrates orthogonal signal correction (OSC) and supervised PLS-DA methods to decompose the X matrix into Y-related and unrelated information, facilitating the selection of differential and characteristic variables [75]. Unlike PCA, OPLS-DA is a supervised statistical method with a focus on the predictive components with enhanced model performance accuracy and reliability of differential and classification analysis. The key distinction from PLS-DA lies in the inclusion of orthogonal correction signals in OPLS-DA, providing filtration of errors caused by non-experimental factors. Internal cross-validation is required to prevent overfitting of the OPLS-DA model [76].

Tatiane et. al. used the Partial Least Squares (PLS) regression technique to predict the presence of fatty acid (C18:1 trans isomer) in chocolate using ^1^H-NMR spectra. External cross-validation was performed when the dataset was split into calibration (training, 70%) and external validation (testing/prediction, 30%). Root mean square error of cross-validation (RMSECV), root mean square error of prediction (RMSEP), and correlation coefficients were used as performance metrics. The results showed a strong correlation and low prediction error (RMSEP = 0.38 g/100 g fat) for total TFA prediction [77]. Lee et. al. utilized Canonical Discriminant Analysis (CDA) to differentiate red pepper powders from different countries (Korea, China, and Vietnam) based on significant metabolites identified from ^1^H NMR data [43]. ^1^H NMR spectroscopy was used for identifying lard adulteration in butter and analyzed using PLS regression, achieving a high regression coefficient (R^2^) with low calibration and prediction errors. The model was tested with cross-validation for the effective predictive power of the model [47]. Honey adulteration was detected based on geographical origin utilizing supervised techniques like PLS-DA, demonstrating superior performance, with exceptional prediction accuracy for key discriminatory variables like sugars, amino acids, and aromatic compounds. Cross-validation was employed to ensure the robustness and predictive ability, confirming that supervised models provide reliable classification compared to traditional manual methods [78].

Further, unsupervised PCA and supervised PLS-DA models effectively classified bovine, goat, and soy milk and distinguished between authentic and adulterated milk [73]. PCA-LDA models were employed to distinguish fresh fish from thawed as well as frozen fish varieties. Six classification models were cross-validated using a repeated Monte Carlo resampling method. Based on the lipid fractions and components, the top two models achieved accuracy rates of more than 90%. External validation was also performed with additional fish samples, which strengthened the robustness of the model for species authenticity [79].

**Data Preprocessing for Multivariate Statistical Analyses:** Despite the advantages of NMR spectroscopy, it suffers from relatively low sensitivity and overlapping of the signals, which can limit the analysis of low-abundance metabolites [80]. To harness the full potential of NMR and enhance the quality of the data, a uniform, rigorous data preprocessing method for transforming raw spectral data into a refined, interpretable format is necessary for downstream statistical analysis and marker discovery [81]. Preprocessing NMR data involves several key steps, including baseline correction, normalization, peak alignment, scaling, and variable selection, all aimed at removing unwanted noise and artifacts while preserving relevant important information [82].

**1. Baseline Correction:** This step removes or minimizes artifacts and possible baseline drifts from the NMR/MRS spectra, which can affect the accuracy of peak detection and integration [81].

**2. Normalization:** The normalization procedure reduces unwanted variability among samples, facilitating more reliable quantitative comparisons. The various methods of normalization, like total area, probabilistic quotient, or quantile, can be used during preprocessing and pretreatment of NMR data for multivariate statistical analysis [81,82].

**3. Peak Alignment:** The metabolite signals must be aligned across all NMR spectra. There could be positional variations in the signals (chemical shifts) due to instrumental drift or the local environment of the sample [83]. If there are any variations, then peak alignment removes that variability, and comparisons can be made for signals across the samples.

**4. Scaling:** Scaling is used to normalize the analytical data (NMR) by adjusting the variability of each metabolite (variable). This prevents the dominance of variables with large magnitudes (intensities) and ensures that all variables contribute relatively equally to the statistical models being used during the analysis [84].

**5. Data Bucketing or Binning:** Data bucketing (or binning) is a method to divide spectra into small segments of chemical shifts (data reduction) where NMR spectra are divided into smaller chemical shifts (buckets or bins). Further, the area under the curve of the signal within each bucket (bin) is calculated and utilized as a variable for downstream statistical analysis [85].

## 6. Applications of Magnetic Resonance in Food Analysis

NMR spectroscopy has been widely used in analyzing herbal medicines and foodstuffs. Some of the applications are discussed in detail below:

**Assessment of Food Quality and Food Safety:** Food quality and safety are often hampered by adulteration in food products. Adulteration diminishes a food’s ability to meet quality and safety requirements and ultimately leads to health and economic losses. In one of the studies, ^1^H-NMR spectroscopy was utilized to discriminate the geographical origins of red pepper powders in Korea from imported varieties. Discriminant analysis was employed for identifying metabolite variations, tackling issues of fraud, and enhancing consumer trust and product authenticity [43]. A novel NMR method has been employed for detecting peanut adulteration in blends with almonds and walnuts, and N-methyl-4-hydroxy-L-proline (3.05 ppm) was identified as a marker signal for peanuts. A linear regression model was used to evaluate the effectiveness of the methodology for food fraud screening, validating peanut mixtures from different origins, as well as crop years for biological variance [59]. ^1^H-NMR metabolite fingerprinting, combined with PCA and OPLS-DA, investigated the authentication of *Curcuma longa* with *Curcuma manga*. The spectra of 50% adulterated *C. longa* resembled those of pure *C. longa*, but PCA successfully differentiated them, and OPLS-DA provided clear separation. Gradient diffusion NMR and multivariate classification methods identified adulteration in extra-virgin olive oils and identified peanut, soybean, and sunflower oils. Discriminant analysis obtained 98% and 100% accuracy for adulterated and non-adulterated samples, respectively [60]. LF-NMR with chemometrics classified and quantified adulteration in sesame oil with soybean oil. Different concentrations of soybean oil produced distinct T2 relaxation and were classified effectively based on the adulteration levels. These data indicate the potential of NMR for rapid detection of adulteration in the food industry [50]. MRI, being a powerful non-invasive technique, has been employed for assessing the quality of various food products [86]. LF-NMR has been utilized for analyzing moisture (residual water) in aquatic food products. LF-NMR measures the transverse relaxation time (T2) of free, bound, or immobilized water in aquatic food products [87]. LF-NMR has also been applied to assess unrefined frying oils. The T2 values showed a third peak in the T2 distribution of the unrefined frying oils, linked with polymers formed during prolonged frying [88]. LF-NMR revealed different hydrogen pools at dispersion, emulsion, and oleogel, as well as concentration and heating by measuring T2 relaxation time [89].

**Figure 4:**
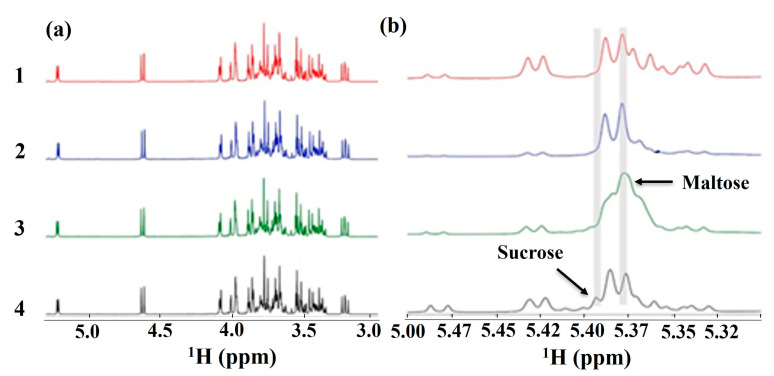
Stacked lot of the ^1^H-NMR spectra of (**a**) full and (**b**) expanded spectra of (1) authentic Indian rapeseed honey and three adulterated samples with (2) brown rice syrup, (3) corn syrup, and (4) jaggery. The chemical shift region from 5.3 ppm to 5.5 ppm shows disaccharides/oligosaccharides, which are key markers for distinguishing authentic honey and adulterated honey samples. Corn- and brown rice-adulterated samples show increased maltose signals, whereas jaggery (4)-adulterated honey shows a prominent sucrose peak [94].

T2 relaxation profiles in muscle revealed three states of water, bound, immobilized, and bulk water, and the T2 of immobilized water remained stable after the first freeze–thaw cycle. T2 decreases significantly after the sixth cycle, indicating water loss in the muscle due to protein denaturation and fiber shrinkage [90,91]. Multiple freeze–thaw cycles reduce T2 relaxation for immobilized water, like that observed for water holding capacity [92]. Saffron (*Crocus sativus*) is very expensive, and it is often adulterated with turmeric or artificial colors. ^1^H-NMR differentiated saffron samples as three natural, one colored with tartrazine, two with colored paper, and eight saffron samples with labeling violations [93]. NMR can play a huge role in food adulteration as it can identify and quantify the adulterants in foodstuffs. Rachineni et. al. demonstrated that ^1^H-NMR spectra clearly show the characteristic peaks for compounds present in authentic Indian rapeseed honey and that adulteration can be easily identified in samples adulterated with rice syrup, corn syrup, and jaggery. The chemical shift region from 5.3 ppm to 5.5 ppm shows characteristic signals for disaccharides/oligosaccharides, which are key markers for distinguishing authentic honey and adulterated honey samples (Figure 4). Corn- and brown rice-adulterated honey samples show increased maltose signals, whereas the jaggery-adulterated honey sample shows a prominent sucrose peak [94].

**Figure 5:**
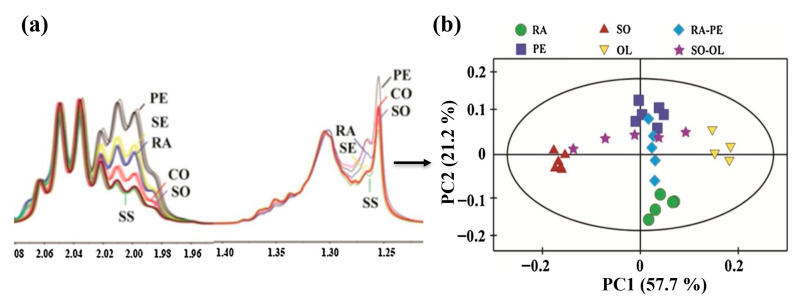
(**a**) Overlapped ^1^H NMR of mixed edible oil: RA (rapeseed oil), PE (peanut oil), RA-PE (mixed oil of rapeseed oil and peanut oil), SO (soybean oil), OL (olive oil), SO-OL (mixed oil of soybean oil and olive oil). (**b**) The principal component analysis (PCA) scores plot shows that ^1^H-NMR data combined with PCA effectively differentiate between pure and mixed edible oils. The position of mixtures near their parent oils confirms their identity and composition. This figure is made up of the two original figures from the cited paper [63].

Richardson et. al. performed ^1^H-NMR spectroscopy with multivariate analysis to detect adulteration in coconut water with sugar solution as low as 1.3%, requiring no sample preparation. NMR showed a linear drift in malate chemical shifts correlated with increased sugar adulteration, with minimal changes in the malate concentration and pH of the solution [95]. ^1^H TD-NMR rapidly detected adulteration in milk with whey, urea, H_2_O_2_, synthetic urine, and synthetic milk at different concentrations (5–50% *v*/*v*). Significant changes were observed in T2 relaxation times with different adulteration levels [14]. Shi et. al. demonstrated that ^1^H-NMR spectra combined with chemometrics can identify authenticated *Citrullus Alatus* oil adulteration. Pure oil was separated from impure oil samples in the PCA scores plot. The responsible variables, like linolenic acid, sitosterol, and fatty acids, were identified as markers for adulteration [51]. In another study, mixed edible oils and their composition were identified by NMR spectroscopy and multivariate statistical analysis (PCA). The scores plot shows that ^1^H-NMR data can effectively differentiate between pure and mixed edible oils [63]. The explained variance by PC1 is ~58%, which demonstrates that the unsupervised PCA model derived from NMR data is able to differentiate between the mixed edible oils (Figure 5).

**Evaluation of Food Processing and Preservation:** MRI has been applied to assess the ripening and mapping of moisture and fat in dairy products, such as quantifying the separation of the cream from milk [96]. MRI has been employed for collecting useful information to analyze the effect of chitosan on the maturity and conservation of citrus [97]. NMR has been instrumental in the characterization of water mobility and distribution of moisture in foodstuffs [98] and investigations of food colloids by NMR and MRI [99]. MRI provides valuable insight into the measurement of storage changes in chocolate confectionery products [78]. Xiuming et. al. determined *cis* and *trans* fatty acid contents in edible oils using characteristic proton regions. The NMR spectra of edible oils and cis/trans isomerized oils differed in regions related to bis-allylic, allylic, and methyl protons. Combined with partial least squares analysis, NMR can be used to analyze the predicted cis/trans fatty acid contents. NMR provides both qualitative (chemical structure) and quantitative information, which makes it an indispensable tool in quality assessment [100].

In conjunction with multivariate analysis, NMR provides a rapid way to quantify adulterants or food supplements. C18:1 trans fatty acid (TFA) isomers were quantified in chocolate fat. The signals from 5.25–5.45 and 1.94–1.99 ppm were crucial for modeling. The novel method utilizes predictive modeling and demonstrates excellent performance for all TFA isomers, highlighting its potential in food quality assessment and fraud detection [77].

**Assessment of Meat and Aquatic Products:** The total fat content and its distribution in meat products are important for taste, texture, and smell. MRI has been extensively used for assessing meat and meat products [101]. Diffusion-weighted MRI, which can be used to measure the diffusion coefficients of protons in water and lipids, has been used to determine the oil uptake by meat during the frying process [98]. Water-holding capacity and interaction of water with the macromolecules determine the nutritional quality of the meat. ^1^H TD NMR measures water distribution in skeletal muscle, assisting in better understanding the mechanisms that enhance meat processing and quality [102]. NMR measures three different relaxation times, i.e., long T2 for the presence of moisture between muscle fibers, medium T2 for myofibrillar proteins, and short T2 represents association with other macromolecules. A decrease in these values reflects moisture and protein losses in the meat product [103]. Combined NMR and MRI techniques can be applied for quantitation of moisture and measuring structure changes during the cooking of chicken meat [104].

Aquatic products, such as fish and seafood, are very popular due to their high-value food therapy, but they can easily oxidize and degrade if proper processing and preservation do not take place. MRI is very helpful in measuring the distribution, size, and shape of the adipose tissues in live fish [105]. LF-NMR and MRI have been utilized in the assessment of aquatic products. NMR has demonstrated its significance in monitoring fish freshness due to its non-invasiveness and multiple NMR parameters for measuring changes in the fish products have been developed over time [106]. MRI has been used to evaluate fat content and measure its distribution in Atlantic mackerel fish under different conditions [107].

^1^H HR-MAS NMR allows rapid determination of indicators of fish freshness: the trimethylamine nitrogen content allows a direct measurement of the quality of unprocessed fish without any extraction [108]. The LF-NMR method was established to monitor the dynamics of protons in fishery products stored at −4 °C for 11 months. The change in the relaxation time T2 with the interaction with macromolecules demonstrated that the moisture migrated through the extracellular spaces, whereas the moisture and fat contents remained unchanged under storage [109]. The composition of other meat types, such as the flesh of brown trout, has also been assessed using MRI [110]. A summary of a number of applications, including methodologies, key findings, and NMR/MRI characteristics from various studies, is shown in Table 1.

## 7. Discussion

**Figure 6:**
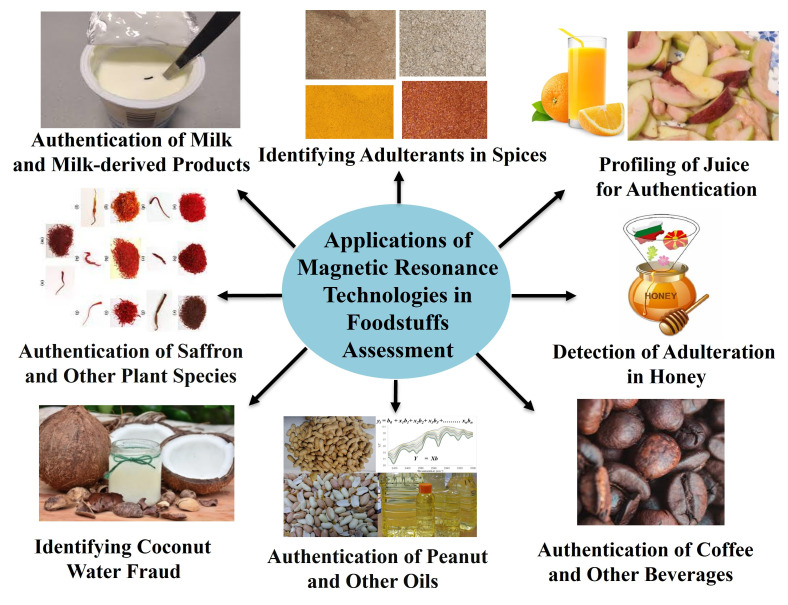
Application of Magnetic Resonance Spectroscopy in analyzing chemical properties of foodstuffs. Magnetic Resonance Spectroscopy offers a non-destructive, detailed analysis of foodstuffs. It helps detect adulterants, monitor spoilage, and profile nutrients and flavors in honey, milk, oils, coffee, coconut water, spices, etc.

Magnetic resonance has been a remarkable scientific discovery that takes advantage of the magnetically active nuclei to characterize molecules in chemical processes and biological systems, making it an indispensable tool for multidisciplinary research. Scientists have been exploring the magnetic resonance-based techniques (MRI and NMR) to understand the dynamic behavior of molecules in various models and assessing the anatomy of living beings [113,114,115]. The application of solution-state, high-resolution magic-angle spinning (HRMAS) and solid-state NMR probes has significantly enhanced the capabilities of NMR technology. Solution-state NMR requires complete solubility of materials in a solvent; otherwise, it will distort signals (the line broadening) due to the magnetic field inhomogeneities. Dissolving all compounds (polar, mid-polar, and nonpolar) in a single solvent is impossible. To avoid this problem, solvent–solvent partitioning (selective extraction) is utilized to isolate similar types of polarity of compounds in a specific fraction that can be adequately dissolved in a single solvent [116,117,118]. It also helps in analyzing the data after the reduction of complexity in NMR spectra. However, the originality of the sample is destroyed as the various solvents are used to treat the samples. The evolution of the magic-angle spinning NMR probe opened many opportunities in material science, biological science, and the food industry. The sample is generally rotated at a frequency of 1000 Hz (depending upon the magnet) at a 54.74° angle with respect to the external magnetic field [31,119]. This innovation helps to reduce the dipolar interaction in solids and reduces the line broadening. Additionally, this technique requires minimal sample preparation as no extraction is required. Low-field ^1^H-NMR relaxometry is an important technique for analyzing the essential components of whole food items using relaxation parameters, self-diffusion of water, and strength of NMR signals. Benchtop NMR has provided information about saffron adulteration by analyzing substances including safflower, turmeric, cayenne pepper, and tartrazine. This could be an alternative approach to the other NMR method. The fraudulent addition of *Robusta* to *Arabica* coffee was also detected via the ^1^H-NMR spectrum of the coffee powder, demonstrating the application of this approach in measuring food adulteration. NMR could be a more reliable tool for detecting food adulteration than traditional techniques. The SIMCA model differentiated pure milk from adulterated milk, highlighting the potential of ^1^H time domain (TD) NMR for milk quality assessment. NMR identifies metabolites like trimethylamine to assess the freshness of fish via measuring the increase in its concentration as fish spoils, and NMR also studies the effects of freeze and thaw cycles on fish quality [120]. Alessandra et al. employed NMR-based metabolomics to observe the effect of different storage temperatures and shelf-life on the metabolic profile of red mullet and bogue fish over time. The alteration in metabolite profile can help in monitoring freshness and quality [121]. NMR has been utilized to differentiate between wild and farmed fish and also to distinguish between different fish species based on their characteristic metabolite profiles [122]. Christina et. al. used NMR to study the impact of diets or environmental factors on the metabolic processes in fish [123]. NMR has been used to detect adulteration in fish products [124] and to study the effects of processing methods on fish [125]. NMR can analyze water distribution and tissue structure to assess the quality and nutritional value of fish [106]. MRI has been used to determine the fat content of fish [98], and ^23^Na MRI has been employed to determine salt distribution in fish [126]. Magnetic resonance technologies (NMR/MRI) have huge potential in identifying adulteration in food and dairy products; authentication of juices, spices, coconut, peanut and other oils; and authentication of beverages (Figure 6). These technologies could be helpful in monitoring the quality, processing, and safety of foodstuffs.

In the gradient-based NMR methodologies, the NMR/MRI probe is heated, and, to prevent probe heating, continuously cooled water is circulated to protect the sample from excessive heating [23]. Diffusion coefficients are calculated using the monoexponential decay of the signal with gradient amplitude. To visualize NMR data more effectively, various multivariate statistical analysis techniques have been developed for investigating complex datasets. Multivariate analysis provides insights into identifying patterns, correlations, and significant differences due to adulteration in various samples. Multivariate statistical analysis involves several models, such as unsupervised PCA, supervised PLS-DA, and hierarchical clustering [127]. These types of visualization tools can reveal the sample clustering, outliers, and adulterants in different conditions. PCA can be useful for identifying the metabolite profiles in different groups (e.g., adulterated vs non-adulterated samples, healthy vs. diseased individuals) [128,129,130,131]. PLS-DA score plots indicate greater separation of samples, revealing a stronger classification effect. The statistical analysis provides insight into identifying markers and understanding adulteration in various samples at various levels [51]. In addition to NMR, several other techniques have been explored for assessment of food quality, authenticity, and adulteration. NMR could be the frontline choice of for researchers as it can quantitate and elucidate the structure of compounds present in foodstuffs. A detailed comparison of the various techniques is depicted in Table 2.

Quantitative Nuclear Magnetic Resonance (qNMR) provides insight into food analysis, precisely quantifies key markers, and verifies food authenticity. qNMR can identify and quantify adulteration in oils, and detect water, synthetic milk, or milk substitutes by analyzing fat and protein contents. qNMR has also been employed for lower-grade substitutes in spices, ensuring authenticity and obtaining good peak alignment. Phase and baseline corrections need to be performed to ensure a better quantitative evaluation of the NMR signals. The choice of internal standard and optimization of T1 relaxation times and optimum recycle delay need to be set during qNMR experiments. In addition to the various types of one-dimensional NMR experiments, two-dimensional NMR (2D NMR) experiments play a crucial role in the chemical profiling of various types of samples. The connectivity in homo- (^1^H-^1^H) and heteronuclear (^1^H-^13^C) correlations in 2D NMR can elucidate structures even in mixtures of various samples. Several 2D NMR experiments like COSY, HSQC, HMBC, TOCSY, NOESY, and HSQC-TOCSY, etc., have been utilized to detect foodstuff adulteration. Two-dimensional NMR experiments are remarkable techniques that have been playing their role in food science to detect the concentration of contaminated substances and identify key metabolites in foodstuffs.

Food adulteration is a longstanding issue that continues to pose significant challenges in the global food production and distribution [132]. A range of analytical techniques, from DNA-based methods to vibrational (IR) spectroscopy, have been employed to detect adulteration in foodstuffs [70,132,133]. There has been a growing recognition of the potential of NMR as a powerful and versatile tool due to its superiority in both structural characterization and quantification [134]. The rapidly evolving food industry has been plagued by the persistent issue of food adulteration. This practice not only compromises the authenticity and quality of food products but also poses significant health risks to consumers. In recent years, developments in NMR technology have provided new avenues for the detection and quantification of food adulterants, making NMR an indispensable and powerful tool in this endeavor [135]. NMR spectroscopy offers several advantages over traditional methods in the analysis of food adulterants. Unlike many other techniques, NMR is a non-destructive, rapid, and highly reproducible method that can provide comprehensive information about the chemical composition of a sample without the need for extensive sample preparation [132,134]. The ability to generate detailed chemical profiles, or fingerprints, of food samples allows for the identification of subtle differences between authentic and adulterated products, enabling the detection of even small amounts of adulterants [134,135]. In addition to NMR, MRI has been recognized as a magnificent tool for assessing the quality of foodstuffs, allowing internal images of foodstuffs to be captured noninvasively and non-destructively [25]. The MRI technique provides images that yield key information about several processes such as alteration in food composition, crystallization, water mobility, food stability, and maturation [136,137].

### 7.1. Advantages of Magnetic Resonance Techniques in Foodstuffs Analysis

**Non-destructive analysis:** NMR and MRI can analyze samples without damaging them, allowing for multiple analyses or preservation of the original sample [138].

**Structural information and inherent resolution of Nuclei (^13^C, ^19^F, and ^31^P):** NMR provides detailed structural information, making it suitable for characterizing and quantifying adulterants and markers with high accuracy [139]. Some NMR active nuclei possess a huge dispersion in chemical shifts, which helps in determining the complex structures in foodstuffs.

**Complex mixture analysis:** Multidimensional NMR experiments excel in analyzing complex mixtures, allowing for simultaneous detection and quantification of multiple adulterants/markers in foodstuffs [140].

**Minimal sample preparation:** Solution-state NMR technique requires sample preparation, but some of the techniques, such as HR-MAS and MRI, require minimal sample preparation, making them relatively easy-to-use techniques [141,142]. Ciampa et al. highlighted that, in sample preparation, NMR avoids use of the hazardous solvents/reagents commonly required in other analytical techniques such as HPLC. NMR can quantify trimethylamine in fish without needing solvents like toluene or formaldehyde, or dangerous reagents such as picric acid. This not only makes the process safer but also reduces environmental impact and improves laboratory sustainability [143].

### 7.2. Disadvantages of Magnetic Resonance Techniques in Foodstuffs Analysis

**High cost:** NMR instruments and their maintenance can be expensive, limiting their accessibility to a wide range of users, and even in remote areas for food quality assessment [144]. Compared to the NIR spectroscopy, the cost of NMR is significantly high. A detailed comparison of both techniques is shown in Table 3.

**Sensitivity limitations:** NMR may have difficulty detecting very low concentrations (up to ppm-level concentrations like Mass Spectrometry) of adulterants, but using a higher magnetic field and increasing the amount of the sample can overcome this problem [139]. For peanut adulteration in various food products, the approximate NMR detection limit on 400 MHz was around 4% concentration of the adulterant [59]. ^1^H-NMR achieved limits of detection between 0.31% and 0.86% for adulterants like barley, corn, and wheat in coffee samples [145]. ^1^H-NMR spectroscopy was able to detect the adulterant Sudan I dye at around 6.7 mg/kg in solution-state NMR in paprika powder [146]. The detection limit for adulterant (sugars) was about 1.3% using ^1^H-NMR in fresh coconut water [95]. Although NMR is less sensitive than other techniques like Mass Spectrometry, recent advancements in NMR magnets and probes have increased the capability of NMR to detect adulterants at sub-percent levels in complex foodstuffs.

**Lack of chemical database of foodstuffs:** There are no databases available for the chemicals present in foodstuffs. Deconvoluting the NMR data and an AI-assisted approach can help in detecting adulterants in foodstuffs [147].

**Complexity of analysis:** In some samples, complex mixtures analysis is difficult due to the large size of molecules present, which can lead to crowded spectra, making analysis challenging [148].

**Not suitable for all food matrices:** NMR and MRI may not be effective for certain solid/semi-solid samples or very heterogeneous systems [149].

### 7.3. Limitations of NMR Applications in Food Science

**1. Sensitivity and Detection Limits:** NMR often requires relatively high concentrations of analytes for accurate detection. ^1^H NMR is relatively sensitive but hampered by low resolution and overlapped signals. Other NMR active nuclei like ^13^C have better resolution, but sensitivity is quite low due to the low natural abundance and low gyromagnetic ratio (magnetic behavior of NMR active nuclei). These factors limit its application in foods with low concentrations of compounds such as trace contaminants and minor constituents (flavors, preservatives, or additives) [150]. However, due to recent advancements in microcoils (or NMR flow cells) and high magnetic fields, the detection limit can be very low in NMR, and helpful in detecting low-abundance compounds in the micromolar (µM) range [151,152,153].

**2. Sample Preparation and requirement of variable MRI coils (resonators):** Solution-state NMR typically requires relatively clean and specific sample preparations, i.e., buffer to mimic the pH of samples with original states. In food science, the complexity and variability of food matrices require rigorous extraction procedures, which can make sample preparation time-consuming and sometimes challenging. Selective methods need to be developed [116,154]. Due to the variable textures, shapes, and sizes of the food products, sometimes it is difficult to fit them into the same MRI coils for image acquisition. This limits the frequent use of MRI for food internal image analysis. The development of microscopic coil-based microscopic MRI (µMRI) methods is also needed for assessing the food quality of small food samples [23,24,155].

**Simplification of operating procedures:** Benchtop NMR spectrometers are designed to be more user-friendly with automated data acquisition. Integration with advanced chemometric software can enhance data processing capabilities, making the technology more accessible to non-specialist users [156].

**3. Cost and accessibility of the instruments:** High-field NMR and MRI instruments, which provide the best results in terms of sensitivity, resolution, and high-throughput analysis, are expensive and not always readily available in every food testing laboratory and R&D institution. This limits the widespread adoption of NMR/MRI for routine analysis in food industries, especially in developing and underdeveloped countries [144].

**Development of Low-Cost NMR Instruments:** Recent advances in NMR technology have produced benchtop or low-field NMR instruments which are more affordable, but these instruments are inferior in terms of sensitivity, resolution, and wide applications compared to higher magnetic fields [157]. Additionally, software-defined radio (SDR) technology has created the possibility of building NMR setups at very low cost for educational and basic research applications [21].

**4. Inherent low-resolution of NMR in detection of complex food mixtures:** Food samples are composed of different compounds (small and large), including proteins, polysaccharides, lipids, amino acids, sugars, and organic acids. The spectral resolution of NMR is greatly hampered by the faster relaxation of the macromolecules, making signal identification much more challenging due to the presence of line broadening caused by prevalent macromolecules in food matrices. Food samples typically have more spectral overlap, which makes NMR analysis complicated, i.e., difficulty in integration and assignment of signals [126]. To overcome these limitations, extraction methodologies need to be optimized for global sample analysis.

**Use of chemometrics and machine learning:** The integration of chemometrics tools can handle complex NMR data, enabling qualitative and quantitative analysis, improving reproducibility with high-throughput analysis, and reducing the need for expert interpretation [158].

**Table 3:** Comparison of NMR Spectroscopy with Near-Infrared (NIR) Spectroscopy in food analysis.

Aspect	NMR Spectroscopy	NIR Spectroscopy
Cost	High initial cost [144].	Generally lower cost instruments; more affordable for routine use [159].
Operation complexity	Requires specialized training for complex data analysis [160].	Easier operation with minimal sample preparation and rapid results [161].
Data Processing	Complex NMR spectra of foodstuffs needing chemometrics with high expertise [162].	Uses multivariate analysis, but spectra are simpler to interpret [161].
Sensitivity and Specificity	High molecular specificity; distinguishes molecular structures and bonds precisely [163].	Provides chemical fingerprints but less molecular specificity and relies on calibration models and standards [164]
Sample Preparation	Usually requires minimal sample preparation and is non-destructive for solid/liquid samples [165].	Minimal-to-no sample preparation and non-destructive [166].
Applications	Quantitative and qualitative analysis of complex food matrices, origin, adulteration, and lipid profiling [167].	Rapid quality control, moisture, fat, protein, sugar content, and adulteration screening [168].
Portability	Benchtop models are available but generally less portable [169].	Portable handheld NIR devices are widely available [170].

### 7.4. Potential Opportunities and Future Prospects in NMR/MRI for Food Analysis

• **Development of NMR-Based Hyphenated Technology:**

Future advancements are much-needed to integrate NMR with complementary analytical technologies, enhancing the synergistic use of hyphenated technologies to combat various research problems in the food industry. Various research groups have made significant advancements in integrating liquid chromatography and mass spectrometry with NMR to create LC-MS-NMR, LC-NMR, and NMR-MS hyphenated technologies [151,171,172,173,174]. These approaches can provide comprehensive chemical profiling by combining the structural elucidation power of NMR with the very high sensitivity and selectivity of MS and the separation efficiency of chromatography. Additionally, these integrated approaches are particularly valuable in the analysis of food matrices, where single techniques may not capture the full metabolomics and lipidomics landscapes.

• **Technological Development in Portable and Benchtop NMR Devices:**

The evolution of compact low-field NMR instruments holds a lot of promise for real-time food quality monitoring with low maintenance costs, but, due to the smaller magnet size, the resolution and sensitivity are not sufficient for mixture analysis. Further advancements in NMR probe technology, like introducing superconducting probes, microcoils, and sensitive multinuclear probes (like ^13^C, ^31^P, ^2^H, etc.) [113,117,175,176], would enhance the monitoring of food processing, detection of food adulterants, and identify compositional changes. Future research and development should focus on improving the resolution and sensitivity of low-field NMR, and user-friendly interfaces for non-specialist users.

• **Application in Food Nanotechnology:**

The utilization of NMR in food nanotechnology provides an emerging and promising opportunity in characterizing nanostructured materials and studying their interactions with food matrices/fluids and biological systems [141]. Future research should prioritize the development of NMR methods to measure the dynamic processes at the nanoscale, and their safety and bioavailability in foodstuffs.

• **Compressed Sensing and Digital Integration with NMR/MRI:**

MRI is sometimes hampered by long acquisition times, which can lead to artifacts in the images. Compressed sensing (CS) is an emerging method that facilitates image reconstruction from partially collected sparse data, reducing image acquisition time significantly by minimizing the requirement of data collection [177]. Recently, deep learning has emerged as a powerful tool for improved and faster reconstruction of MRI via integration with CS. Incorporating machine learning (ML) for AI-driven data analysis with NMR can significantly enhance data interpretation and analysis in large-scale foodomics studies [178]. Automated peak identification, spectral deconvolution, and predictive modeling can make NMR more accessible and efficient [147].

• **NMR and MRI Databases for Rapid Analysis:**

Development of standardized protocols and expanding spectral and imaging libraries for foodstuffs could facilitate cross-laboratory comparisons and data reproducibility [179]. The work in this direction could also aim to curate NMR/MRI databases for various food types, species and geographical origins to support food quality, food authenticity, and adulterant traceability.

## 8. Conclusions

Magnetic resonance techniques such as NMR and MRI can play a huge role in the quality assessment of various foodstuffs. These techniques can provide qualitative and quantitative information on markers in pure and adulterated samples. Minimal pretreatment is required for solid-state and foodstuff imaging, making these techniques remarkable for quality assessment. The imaging techniques have huge potential as they could be exploited for analyzing foodstuffs in healthy and diseased conditions. The timely monitoring of quality parameters may increase food quality, improve food processing, and enhance the production economy. The application of LF-NMR and MRI can be further explored globally in food processing, such as in drying, freezing, fermentation, and internal quality evaluation. The markers responsible for diseased conditions can be identified with magnetic resonance technologies. To enhance the potential of NMR in this field, a new technique such as LC-MS-NMR [151,180] could be developed for simultaneous profiling and identification of new markers for quality assessment of food products. NMR is a powerful technique in the realm of food authentication, offering comprehensive insights into the overall composition of food products. While NMR spectroscopy excels due to its non-destructive capability and simultaneous detection of multiple chemical components, its application in food authentication has some limitations [181]. The cost of NMR spectrometers, especially those with high magnetic field strengths, can be a significant barrier for some laboratories/universities, hindering widespread adoption of this technique. The maintenance of NMR magnets often requires cryogenic liquids (i.e., helium and nitrogen), which adds to the operational expenses and logistical complexities [182]. The complexity of NMR spectra due to heterogeneous food matrices can pose significant challenges in data interpretation and analysis, requiring deconvolution approaches and computational tools to model the spectra and gain meaningful results [183]. Time-consuming and high-throughput screening is also a challenge, and requires advancements in NMR to make it possible to solve a wider range of analytical tasks using automation in NMR. NMR is a relatively lower-sensitivity technique compared to mass spectrometry techniques. This can be problematic when dealing with important trace components/adulterants with very low concentrations in food samples [181]. NMR spectroscopy holds great potential in food metabolite profiling, quality assessment, and verifying food authenticity. High instrument costs and limited portability restrict the widespread adoption of NMR in routine food testing. The design and development of cost-effective and portable NMR instruments would greatly enhance accessibility for on-site and industrial applications. Integrating NMR with other analytical techniques such as mass spectrometry and chromatography could improve metabolomic coverage, allowing for a more comprehensive understanding of food matrices. The application of machine learning and chemometrics to complex NMR data could simplify interpretation, classification, and predictive modelling in food authenticity and safety. The wide utilization of NMR technology could promote interdisciplinary collaborations in emerging areas such as personalized nutrition, food nanotechnology, food authenticity, and sustainability evaluation. There is a pressing need to overcome the current challenges in NMR and convert challenges into opportunities. NMR could evolve as an important tool for enhancing food safety, quality, and traceability globally.

## Figures and Tables

**Table 1:** Summary of some of the applications of NMR/MRI in food authentication and detection of adulterants for food quality assessment.

S. No.	Study Carried Out	Key Findings	Conclusion	Ref. No.
**1**	NMR spectroscopy was utilized to assess the quality of black pepper (*Piper nigrum*) samples originating from India, Vietnam, and Pakistan.	Pakistani black pepper had the highest piperine content; the Vietnamese sample was superior in overall quality.	^1^H and ^13^C NMR data confirm the presence of piperidine, amide, and pyrrolidine derivatives, whereas IR shows the presence of peaks and features that resemble the standard drug piperine.	[68]
**2**	Truzzi et al. used ^13^C NMR spectroscopy to detect and identify vegetable oil (VO) adulterants in essential oils (EOs).	Palmitic acid, oleic acid, and linoleic acid were identified as chemical markers in the triglycerides of VOs.	For identification of adulterants in oil, ^13^C NMR was preferred over ^1^H-NMR due to the ease of interpretation. Indeed, ^1^H-NMR of Vos exhibited several overlapping signals with poor spectral resolution.	[111]
**3**	Bertelli et al. utilized NMR spectroscopy with chemometrics to detect honey adulteration by intentionally adding sugar syrups.	Sugars (maltose and glucose) are identified as key markers for detecting honey adulteration.	NMR allows discrimination between the signals of very similar chemical structures, like sugars, and allows simultaneous quantification with precision and accuracy, but FTIR is a less informative technique for discriminating sugars.	[61]
**4**	Bertram and Andersen’s group employed NMR relaxometry to study water distribution and mobility in meat.	Water mobility is identified as a physical marker; lactic acid, amino acids, and lipids/fatty acids are identified as chemical markers.	NMR can measure the diffusion of the water molecules and fiber orientation in meat samples, which is not possible using mass spectrometry and IR.	[90]
**5**	Cai et al. used ^1^H-NMR spectroscopy for the quality evaluation and adulteration identification of edible oils.	Linolenic acid, oleic acid, triglycerides, and 1,3′-diglycerides identified as key markers.	^1^H-NMR is a sensitive, fast, and convenient tool for the identification of adulterants and quality assessment of edible oils.	[63]
**6**	^1^H-NMR spectroscopy combined with multivariate data analysis was employed to detect butter adulteration with lard, and Partial Least Squares (PLS) regression was used to quantify lard content in butter samples.	Free fatty acids and fatty acids attached to glycerol, as well as the glycerol backbone, identified as chemical markers.	^1^H-NMR and chemometrics could be a rapid, non-destructive, and powerful tool for the authentication of dairy foodstuffs.	[47]
**7**	^1^H-NMR spectroscopy with multivariate statistical analysis (PCA and LDA) was employed to classify and authenticate different grape varieties in Chinese wines, both red and white.	Sugars, organic acids, ethanol, volatile compounds, and phenolic compounds identified as chemical markers.	Demonstrated the potential of NMR with multivariate models for verifying the different grape varieties in Chinese red and white wines, expanding its application in the liquor sector.	[69]
**8**	NMR in conjunction with multivariate analysis was employed to discriminate between two species of cinnamon: *Cinnamomum verum* and *Cinnamomum cassia.*	Eugenol and fatty acids have been identified as reliable markers for differentiation.	NMR fingerprinting of cinnamon resources, providing an examination of sensory characters of the barks. Volatiles and primary metabolites were identified and quantified using ^1^H-NMR, providing novel insight into their phytoconstituents.	[62]
**9**	MRI was used to investigate the use of chitosan, a biopolymer, as a preservative and fungistatic agent for citrus fruits, specifically Fortune mandarins and Valencia oranges.	Water distribution and its mobility in the fruit were identified as key markers for imaging.	The dissolution of chitosan in the fruits produced excellent results in terms of weight loss and visual appearance. MRI monitors the process of fruit ripening and decay.	[97]
**10**	^1^H-NMR spectroscopy with chemometric analysis was employed to assess the traceability and authentication of “Tuscan PGI” Extra-Virgin Olive Oils (EVOOs).	Fatty acids (oleic acid, saturated fatty acids, polyunsaturated fatty acids) are identified as chemical markers.	The results of this work confirmed the requirement of monocultivar genetically certified samples for constructing a ^1^H-NMR-based metabolic database for cultivar and/or geographical variation.	[45]
**11**	The study used 60 MHz ^1^H-NMR spectroscopy (low-field benchtop proton NMR) to authenticate saffron and detect potential adulterants.	Picrocrocin, crocins, fatty acids, and kaempferol are identified as primary markers in saffron.	Low-cost, low-risk, and suitable extraction method for 60 MHz benchtop NMR, providing spectra with clear features of secondary metabolites in saffron samples.	[19]
**12**	NMR spectroscopy was utilized to authenticate coffee blends by 16-O-Methylcafestol. This study was employed to distinguish Robusta coffee beans from Arabica beans.	16-O-Methylcafestol is identified as a useful marker for distinguishing Robusta coffee beans from Arabica beans.	NMR chemical shifts provide information about the esterified and non-esterified compounds in the mixture. The shifting of peaks indicates the degradation of the food products.	[20]
**13**	Mannina et al. developed a method employing ^1^H-NMR spectroscopy to detect the adulteration of refined olive oil with refined hazelnut oil.	Linolenic acid, squalene, palmitic and stearic residues, and β-sitosterol were identified as chemical markers.	^1^H-NMR shows potential to detect adulteration of olive oil with hazelnut oil at low levels (10%). NMR does not require extraction and can be used to detect olive oil adulteration. Compared to other spectroscopic techniques, it does not have problems in signal quantification for both major and minor components present in olive oils.	[12]
**14**	^1^H-NMR spectroscopy has been utilized to investigate the adulteration of fresh coconut water. The adulteration was carried out with water–sugar mixtures.	Malic acid signals were identified as a potential marker for detecting adulteration in coconut water.	^1^H-NMR spectroscopy enables quantification of the degree of adulteration. The chemical shift and lineshape of malic acid can be utilized as a potential marker for the substitution of coconut water with extrinsic components.	[95]
**15**	HRMAS NMR spectroscopy and multivariate analysis hold tremendous potential for the characterization of adulterants in Italian sweet pepper (*Capsicum annuum* L.).	Sugars (glucose, fructose, sucrose), organic acids (malate, ascorbate, acetate), amino acids (e.g., glutamine, threonine, GABA), and fatty acids (both saturated and unsaturated) identified as chemical markers.	HRMAS NMR provides information about amino acids, organic acids, fatty acids, and other metabolites, without any extraction and purification. HRMAS NMR with PLS-DA proved to be a very useful tool in food science and can be applied to any foodstuff.	[49]
**16**	Low-field Nuclear Magnetic Resonance (LF-NMR) and chemometric methods were employed to detect sesame oil adulteration with soybean oil.	Shorter relaxation time corresponding to protons in linoleic acid and longer relaxation time corresponding to protons in oleic acid were identified as key parameters.	The T2 relaxation time is a robust diagnostic parameter for identifying adulteration, as it depends upon the relaxation of the different types of protons in a specific environment, and adulteration causes a change in the T2 relaxation time.	[44]
**17**	^1^H TD-NMR combined with multivariate analysis was employed to detect and quantify milk adulteration. Various adulterants, including hydrogen peroxide, synthetic urine, whey, urea, and synthetic milk, were introduced to milk samples.	The T_2_ relaxation times of the milk were identified as a chemical marker that was associated with the adulterant concentrations.	^1^H TD-NMR combined with chemometrics may be used for the automation of milk analysis with high-throughput screening without any sample preparation.	[14]
**18**	An MRI technique was used to compare the internal morphology of six new kiwifruit selections with the well-known “Hayward” cultivar.	T_2_ relaxation times (longer T_2_ linked with ripening tissue and shorter T_2_ linked with less ripe tissue) were identified as a robust parameter.	MRI is a powerful imaging tool in assessing food quality, providing images about the spin density (mainly water) distribution, and the relationship between water and its binding cellular tissues.	[29]
**19**	^1^H, ^13^C, and ^31^P NMR techniques were employed to analyze the free fatty acids (FFA) in vegetable oils, such as waste cooking oils (WCO).	Palmitic acid, stearic acid, oleic acid, linoleic acid, and α-linolenic acid were identified as chemical markers.	The advantage of the chemical shift dispersion in ^31^P NMR is that it provides characteristic signals corresponding to phosphitylated sterols, diglycerides, and fatty acids in virgin olive oil.	[112]
**20**	^1^H-NMR combined with chemometric analysis was employed for profiling and determination of C18:1 trans fatty acids (TFA) positional isomers in chocolate.	Chemical shift values at 5.25–5.45 ppm and 1.94–1.99 ppm were used as markers to predict the amount of TFA isomers in chocolate.	^1^H-NMR determines positional isomers and total TFA content in chocolate. The CH (double bonds and glycerol backbone), and CH_2_ (allylic to trans double bonds) signals were identified for rapid analysis, and may be used for TFA monitoring in the chocolate industry.	[77]

**Table 2:** Comparative analysis of NMR with other techniques in food quality assessment.

Technique	Analytical Capability	Core Strengths	Limitations	Suitable Food Matrices	Applications
(NMR) Nuclear Magnetic Resonance	Non-destructive, reproducible	Comprehensive metabolic profiling, precise structure elucidation	High cost and less sensitive than MS	Dairy, meat, fruits, juices	Adulteration detection, authenticity, metabolic fingerprinting
LC-MS (Liquid Chromatography–Mass Spectrometry)	High sensitivity, separation and identification	Detection of trace compounds	Sample preparation intensive, matrix effects	Grains, oils, beverages	Contaminant analysis, pesticide residues, targeted metabolomics
GC-MS (Gas Chromatography–Mass Spectrometry)	Volatile compound detection	Excellent for flavor profiling	Derivatization often needed	Spices, oils, coffee, processed foods	Aroma compounds, quality control
FTIR (Fourier-Transform Infrared Spectroscopy)	Rapid, non-destructive	Easy to use, cost-effective	Limited to functional group information	Dairy, oils, flour	Composition screening, authenticity
NIR (Near-Infrared Spectroscopy)	Fast, portable, minimal sample preparation	Suitable for routine testing	Lower sensitivity and resolution	Cereals, meat, fruits	Moisture, fat, and protein estimation
UV–Vis Spectroscopy	Simple, economical	Quick quantitative assays	Limited to chromophore detection	Beverages, honey, vegetables	Antioxidant activity, polyphenol content

## Data Availability

No new data were created or analyzed in this study. Data sharing is not applicable to this article.
